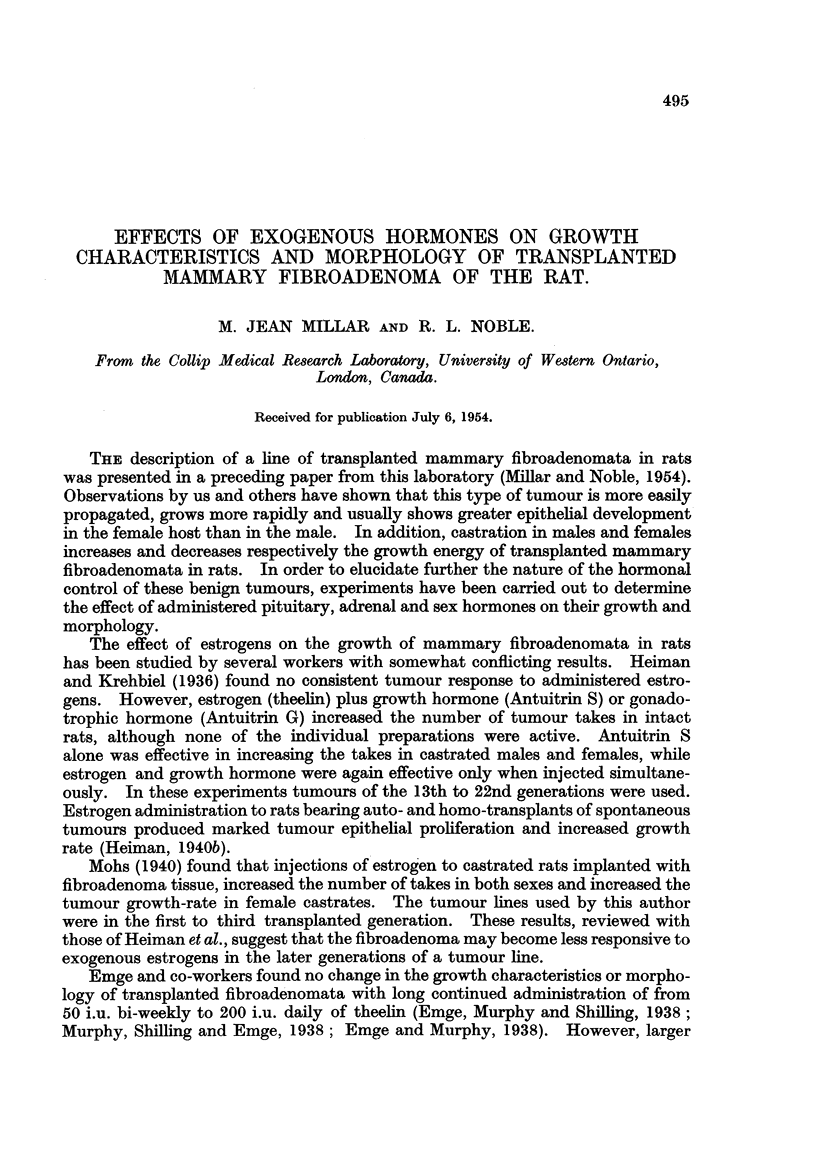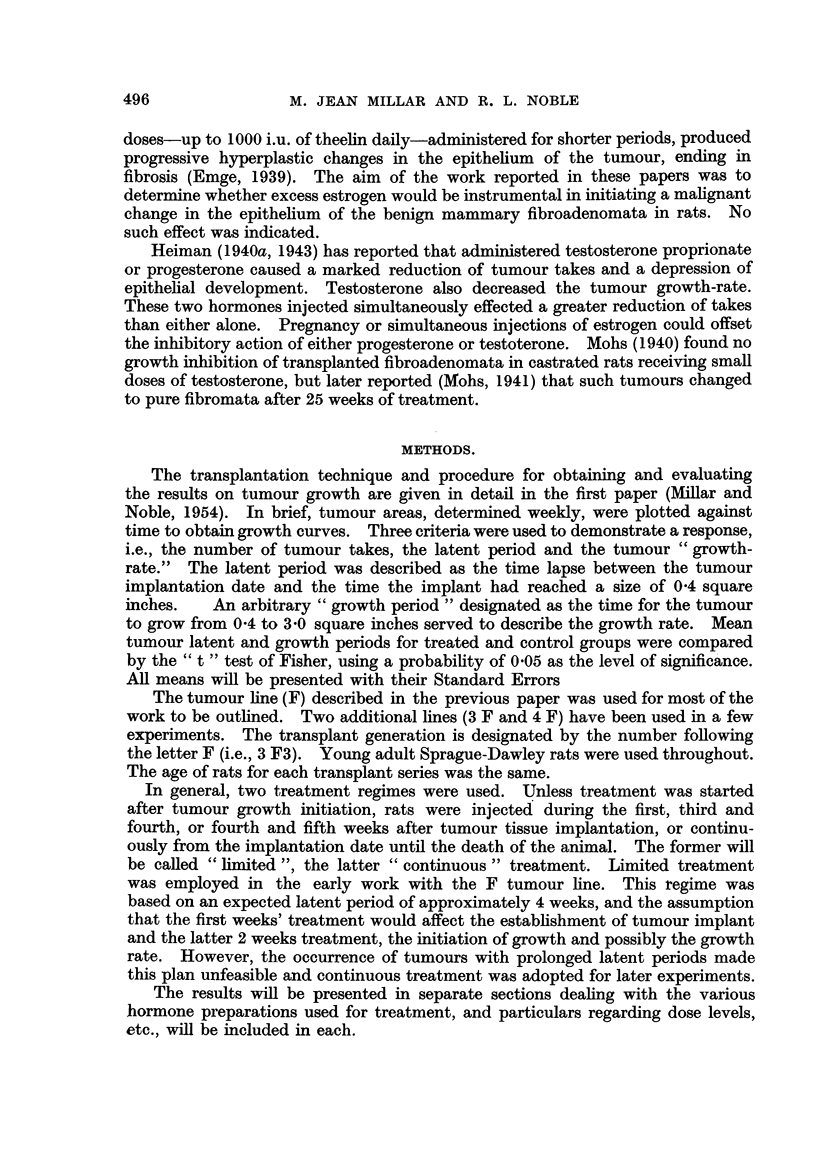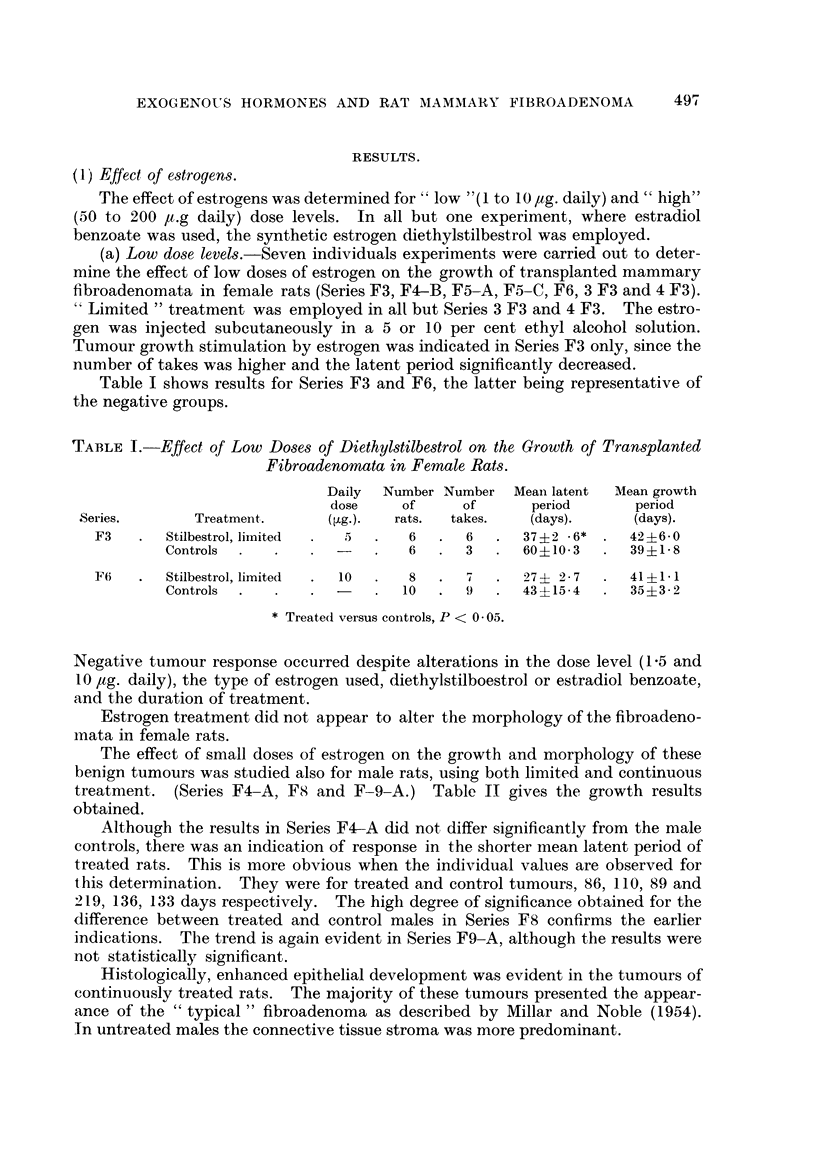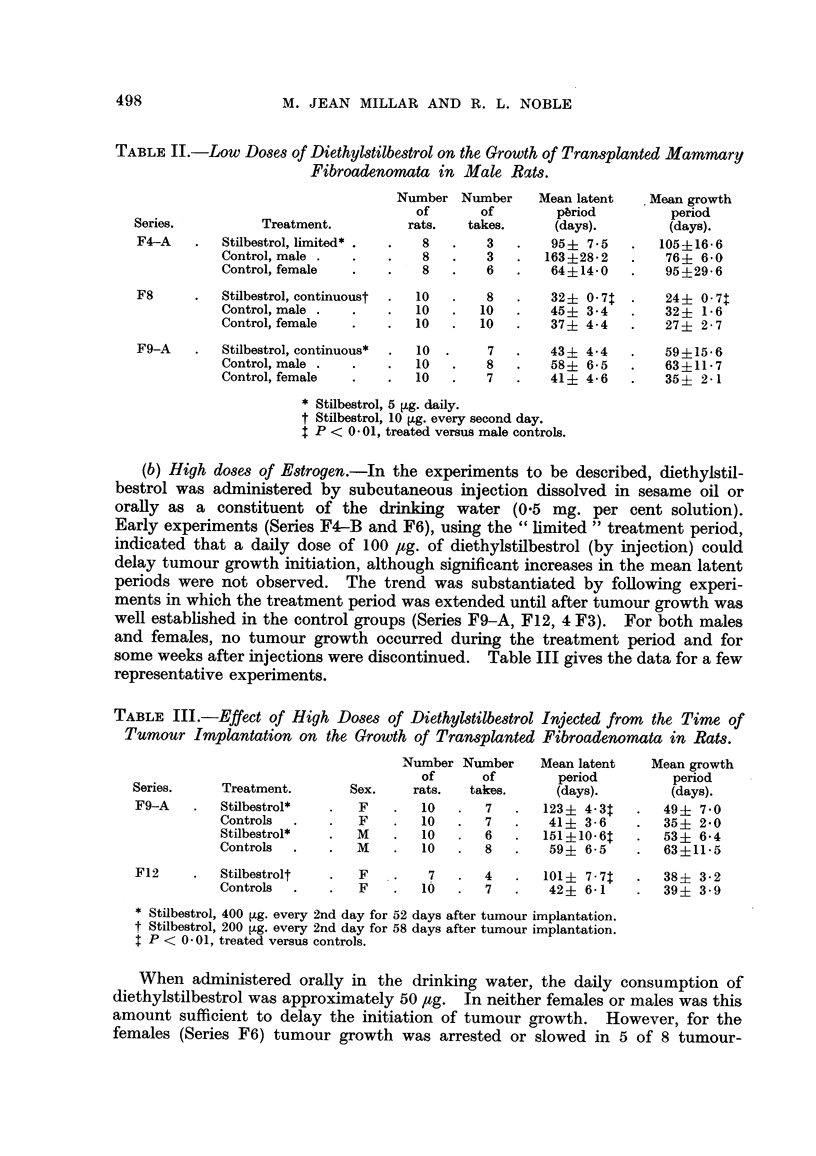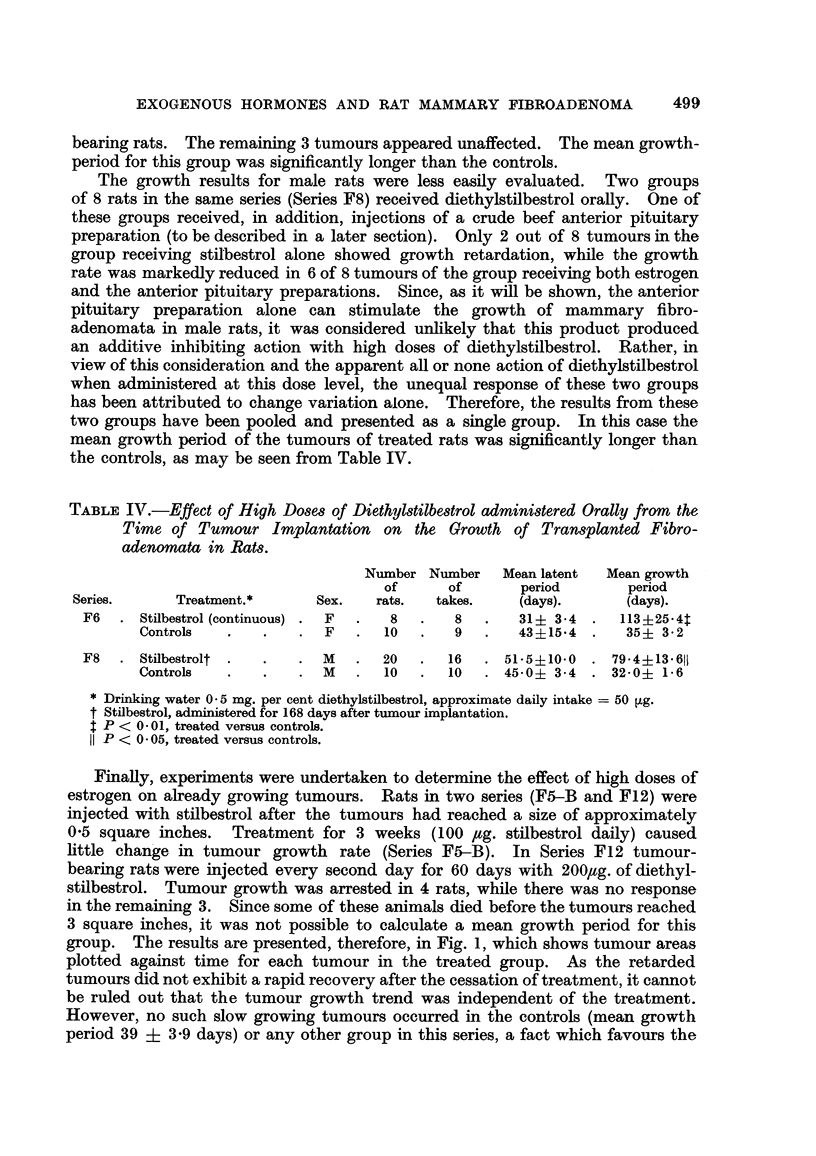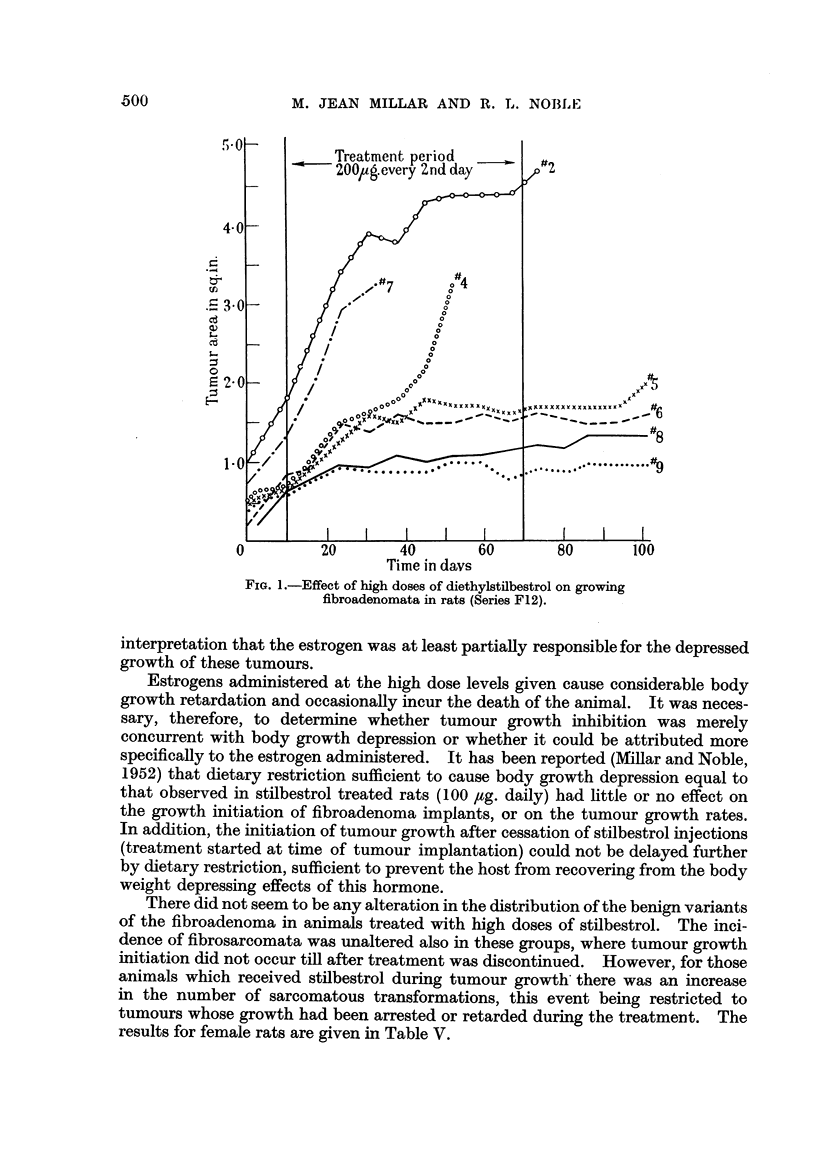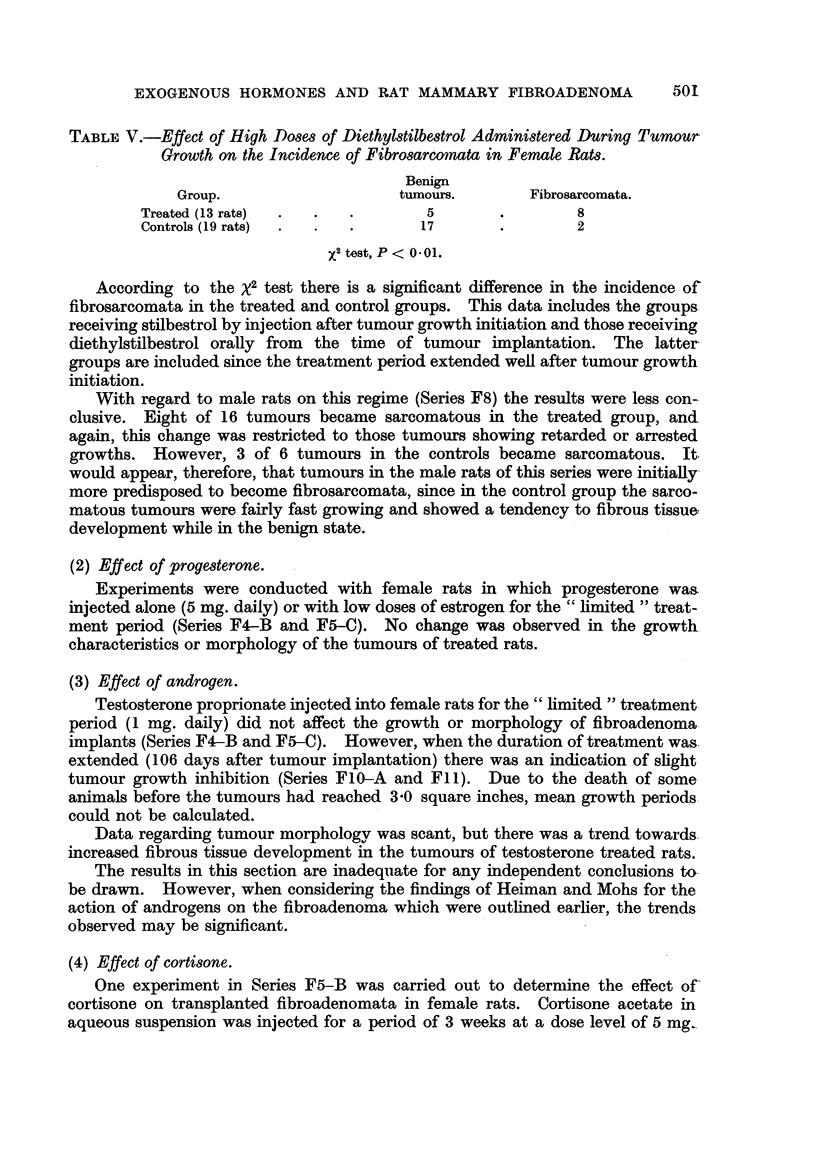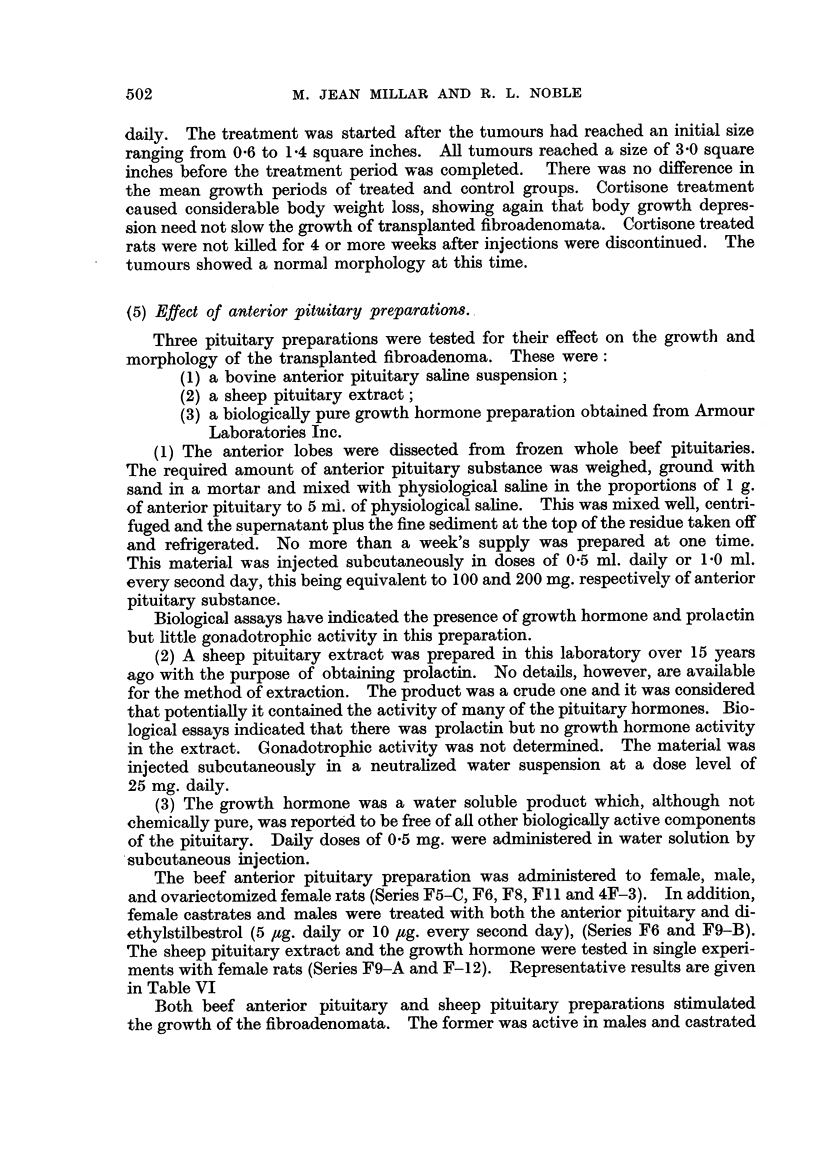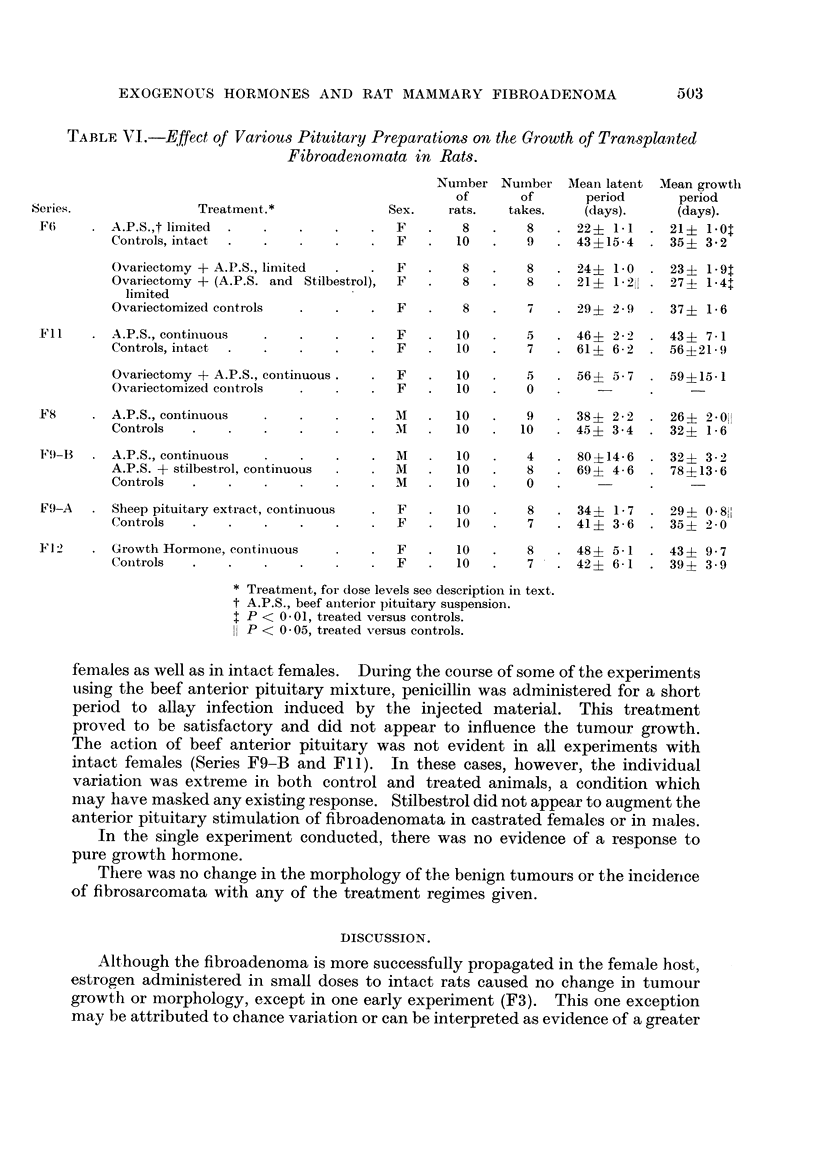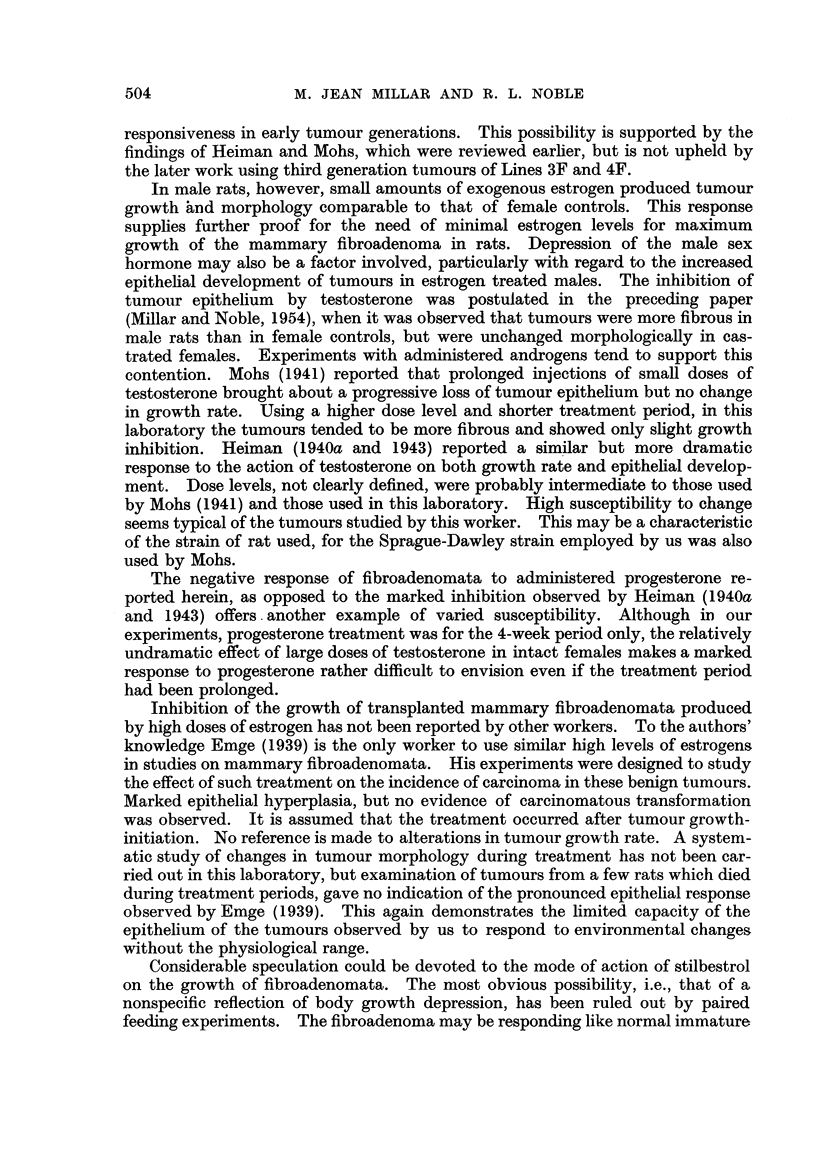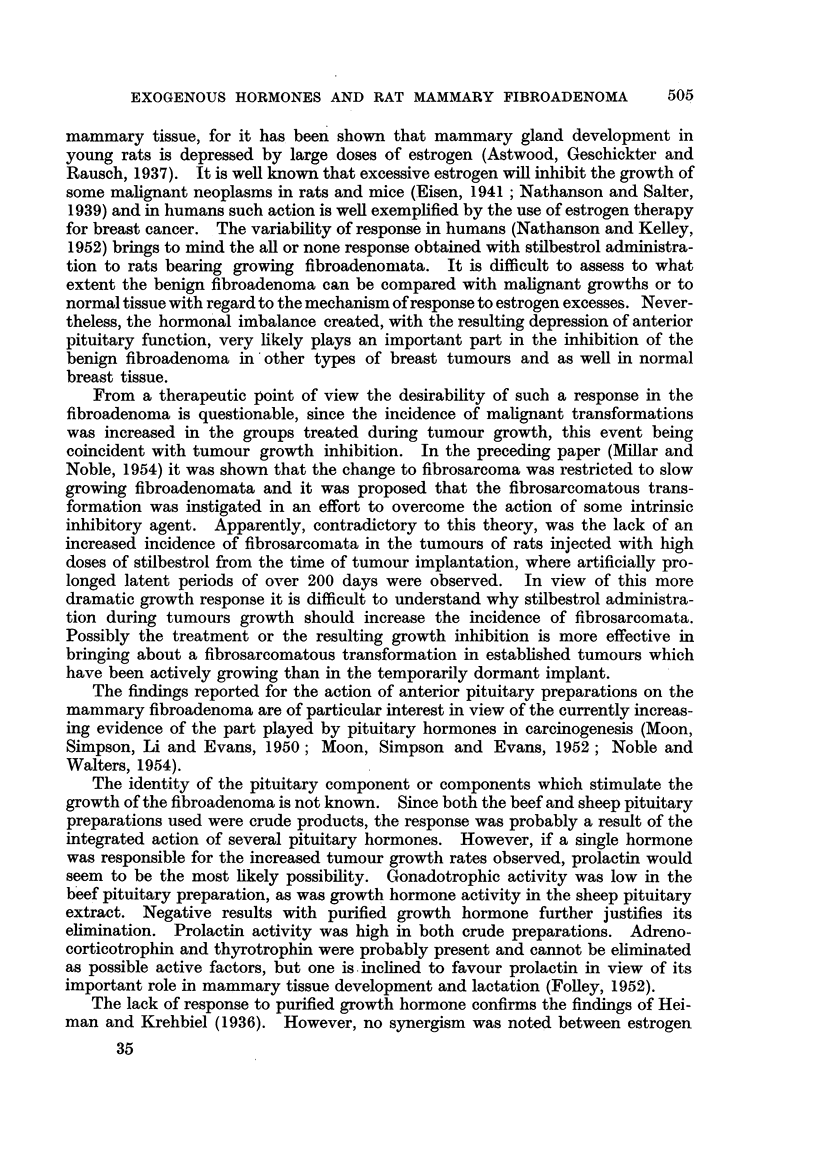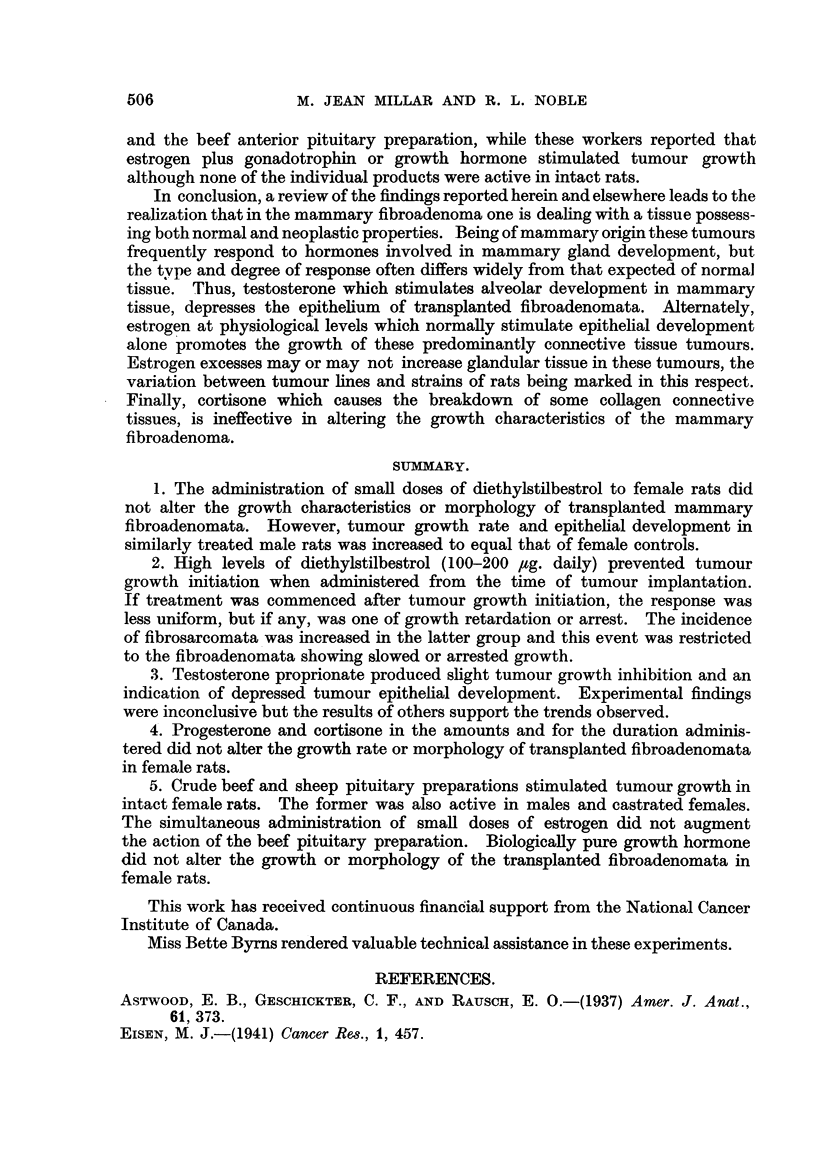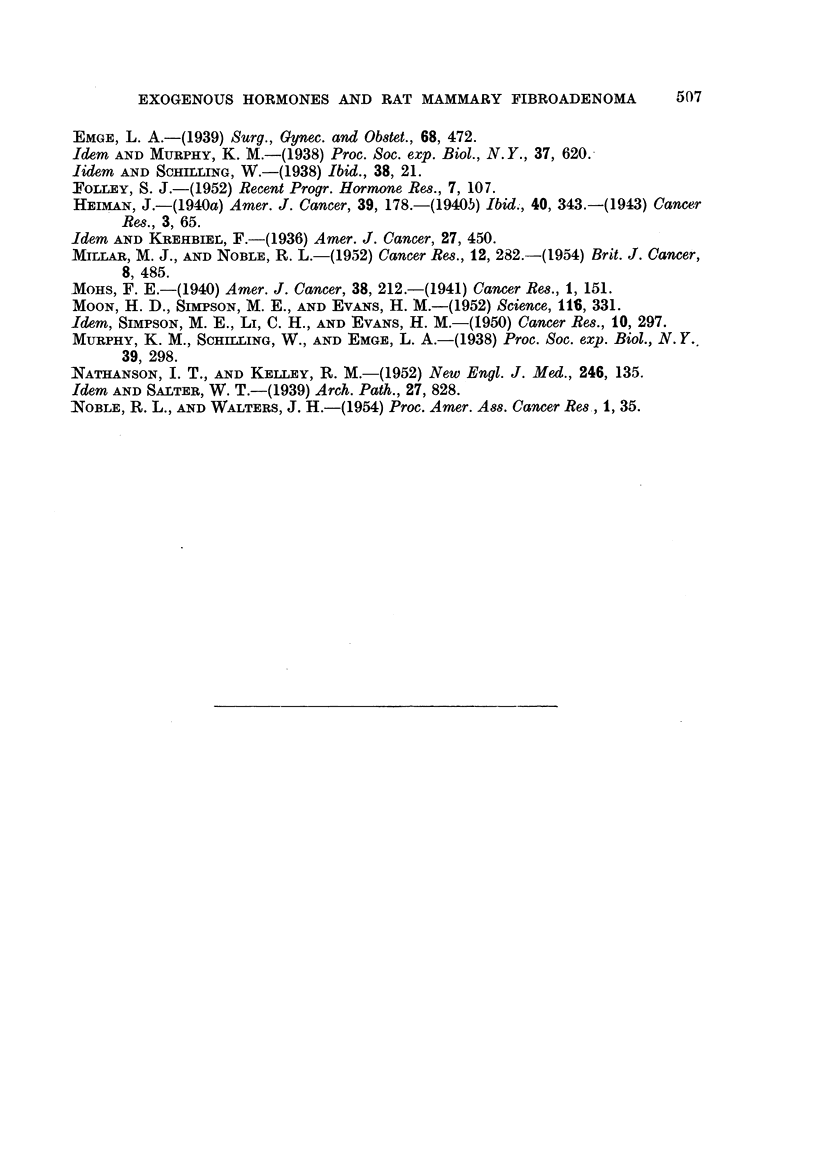# Effects of Exogenous Hormones on Growth Characteristics and Morphology of Transplanted Mammary Fibroadenoma of the Rat

**DOI:** 10.1038/bjc.1954.52

**Published:** 1954-09

**Authors:** M. Jean Millar, R. L. Noble


### EFFECTS OF EXOGENOUS HORMONES ON GROWTH CHARACTERISTICS AND MORPHOLOGY OF TRANSPLANTED MAMMARY FIBROADENOMA OF THE RAT.

M. JEAN MILLAR AND R. L. NOBLE.

*From the Collip Medical Research Laboratory, University of Western Ontario, London, Canada.*

Received for publication July 6, 1954.

THE description of a line of transplanted mammary fibroadenomata in rats was presented in a preceding paper from this laboratory (Millar and Noble, 1954). Observations by us and others have shown that this type of tumour is more easily propagated, grows more rapidly and usually shows greater epithelial development in the female host than in the male. In addition, castration in males and females increases and decreases respectively the growth energy of transplanted mammary fibroadenomata in rats. In order to elucidate further the nature of the hormonal control of these benign tumours, experiments have been carried out to determine the effect of administered pituitary, adrenal and sex hormones on their growth and morphology.

The effect of estrogens on the growth of mammary fibroadenomata in rats has been studied by several workers with somewhat conflicting results. Heiman and Krehbiel (1936) found no consistent tumour response to administered estrogens. However, estrogen (theelin) plus growth hormone (Antuitrin S) or gonadotrophic hormone (Antuitrin G) increased the number of tumour takes in intact rats, although none of the individual preparations were active. Antuitrin S alone was effective in increasing the takes in castrated males and females, while estrogen and growth hormone were again effective only when injected simultaneously. In these experiments tumours of the 13th to 22nd generations were used. Estrogen administration to rats bearing auto- and homo-transplants of spontaneous tumours produced marked tumour epithelial proliferation and increased growth rate (Heiman, 1940*b*).

Mohs (1940) found that injections of estrogen to castrated rats implanted with fibroadenoma tissue, increased the number of takes in both sexes and increased the tumour growth-rate in female castrates. The tumour lines used by this author were in the first to third transplanted generation. These results, reviewed with those of Heiman *et al.*, suggest that the fibroadenoma may become less responsive to exogenous estrogens in the later generations of a tumour line.

Emge and co-workers found no change in the growth characteristics or morphology of transplanted fibroadenomata with long continued administration of from 50 i.u. bi-weekly to 200 i.u. daily of theelin (Emge, Murphy and Shilling, 1938; Murphy, Shilling and Emge, 1938; Emge and Murphy, 1938). However, larger doses—up to 1000 i.u. of theelin daily—administered for shorter periods, produced progressive hyperplastic changes in the epithelium of the tumour, ending in fibrosis (Emge, 1939). The aim of the work reported in these papers was to determine whether excess estrogen would be instrumental in initiating a malignant change in the epithelium of the benign mammary fibroadenomata in rats. No such effect was indicated.

Heiman (1940*a*, 1943) has reported that administered testosterone proprionate or progesterone caused a marked reduction of tumour takes and a depression of epithelial development. Testosterone also decreased the tumour growth-rate. These two hormones injected simultaneously effected a greater reduction of takes than either alone. Pregnancy or simultaneous injections of estrogen could offset the inhibitory action of either progesterone or testoterone. Mohs (1940) found no growth inhibition of transplanted fibroadenomata in castrated rats receiving small doses of testosterone, but later reported (Mohs, 1941) that such tumours changed to pure fibromata after 25 weeks of treatment.

### METHODS.

The transplantation technique and procedure for obtaining and evaluating the results on tumour growth are given in detail in the first paper (Millar and Noble, 1954). In brief, tumour areas, determined weekly, were plotted against time to obtain growth curves. Three criteria were used to demonstrate a response, i.e., the number of tumour takes, the latent period and the tumour " growth-rate." The latent period was described as the time lapse between the tumour implantation date and the time the implant had reached a size of 0·4 square inches. An arbitrary " growth period " designated as the time for the tumour to grow from 0·4 to 3·0 square inches served to describe the growth rate. Mean tumour latent and growth periods for treated and control groups were compared by the " t " test of Fisher, using a probability of 0·05 as the level of significance. All means will be presented with their Standard Errors

The tumour line (F) described in the previous paper was used for most of the work to be outlined. Two additional lines (3 F and 4 F) have been used in a few experiments. The transplant generation is designated by the number following the letter F (i.e., 3 F3). Young adult Sprague-Dawley rats were used throughout. The age of rats for each transplant series was the same.

In general, two treatment regimes were used. Unless treatment was started after tumour growth initiation, rats were injected during the first, third and fourth, or fourth and fifth weeks after tumour tissue implantation, or continuously from the implantation date until the death of the animal. The former will be called " limited ", the latter " continuous " treatment. Limited treatment was employed in the early work with the F tumour line. This regime was based on an expected latent period of approximately 4 weeks, and the assumption that the first weeks' treatment would affect the establishment of tumour implant and the latter 2 weeks treatment, the initiation of growth and possibly the growth rate. However, the occurrence of tumours with prolonged latent periods made this plan unfeasible and continuous treatment was adopted for later experiments.

The results will be presented in separate sections dealing with the various hormone preparations used for treatment, and particulars regarding dose levels, etc., will be included in each.

### RESULTS.

(1) *Effect of estrogens.*

The effect of estrogens was determined for " low "(1 to 10 $\mu$g. daily) and " high" (50 to 200 $\mu$.g daily) dose levels. In all but one experiment, where estradiol benzoate was used, the synthetic estrogen diethylstilbestrol was employed.

(a) *Low dose levels.*—Seven individuals experiments were carried out to determine the effect of low doses of estrogen on the growth of transplanted mammary fibroadenomata in female rats (Series F3, F4–B, F5–A, F5–C, F6, 3 F3 and 4 F3). " Limited " treatment was employed in all but Series 3 F3 and 4 F3. The estrogen was injected subcutaneously in a 5 or 10 per cent ethyl alcohol solution. Tumour growth stimulation by estrogen was indicated in Series F3 only, since the number of takes was higher and the latent period significantly decreased.

Table I shows results for Series F3 and F6, the latter being representative of the negative groups.

TABLE I.—*Effect of Low Doses of Diethylstilbestrol on the Growth of Transplanted Fibroadenomata in Female Rats.*

| Series. | Treatment. | Daily dose ($\mu$g.). | Number of rats. | Number of takes. | Mean latent period (days). | Mean growth period (days). |
|---|---|---|---|---|---|---|
| F3 | Stilbestrol, limited | 5 | 6 | 6 | 37±2 ·6* | 42±6·0 |
| | Controls | — | 6 | 3 | 60±10·3 | 39±1·8 |
| F6 | Stilbestrol, limited | 10 | 8 | 7 | 27± 2·7 | 41±1·1 |
| | Controls | — | 10 | 9 | 43±15·4 | 35±3·2 |

* Treated versus controls, $P < 0·05$.

Negative tumour response occurred despite alterations in the dose level (1·5 and 10 $\mu$g. daily), the type of estrogen used, diethylstilboestrol or estradiol benzoate, and the duration of treatment.

Estrogen treatment did not appear to alter the morphology of the fibroadenomata in female rats.

The effect of small doses of estrogen on the growth and morphology of these benign tumours was studied also for male rats, using both limited and continuous treatment. (Series F4–A, F8 and F–9–A.) Table II gives the growth results obtained.

Although the results in Series F4–A did not differ significantly from the male controls, there was an indication of response in the shorter mean latent period of treated rats. This is more obvious when the individual values are observed for this determination. They were for treated and control tumours, 86, 110, 89 and 219, 136, 133 days respectively. The high degree of significance obtained for the difference between treated and control males in Series F8 confirms the earlier indications. The trend is again evident in Series F9–A, although the results were not statistically significant.

Histologically, enhanced epithelial development was evident in the tumours of continuously treated rats. The majority of these tumours presented the appearance of the " typical " fibroadenoma as described by Millar and Noble (1954). In untreated males the connective tissue stroma was more predominant.

TABLE II.—*Low Doses of Diethylstilbestrol on the Growth of Transplanted Mammary Fibroadenomata in Male Rats.*

| Series. | Treatment. | Number of rats. | Number of takes. | Mean latent period (days). | Mean growth period (days). |
|---|---|---|---|---|---|
| F4–A | Stilbestrol, limited* | 8 | 3 | 95± 7·5 | 105±16·6 |
| | Control, male | 8 | 3 | 163±28·2 | 76± 6·0 |
| | Control, female | 8 | 6 | 64±14·0 | 95±29·6 |
| F8 | Stilbestrol, continuous† | 10 | 8 | 32± 0·7‡ | 24± 0·7‡ |
| | Control, male | 10 | 10 | 45± 3·4 | 32± 1·6 |
| | Control, female | 10 | 10 | 37± 4·4 | 27± 2·7 |
| F9–A | Stilbestrol, continuous* | 10 | 7 | 43± 4·4 | 59±15·6 |
| | Control, male | 10 | 8 | 58± 6·5 | 63±11·7 |
| | Control, female | 10 | 7 | 41± 4·6 | 35± 2·1 |

* Stilbestrol, 5 μg. daily.

† Stilbestrol, 10 μg. every second day.

‡ $P < 0 \cdot 01$, treated versus male controls.

(*b*) *High doses of Estrogen.*—In the experiments to be described, diethylstilbestrol was administered by subcutaneous injection dissolved in sesame oil or orally as a constituent of the drinking water (0·5 mg. per cent solution). Early experiments (Series F4–B and F6), using the " limited " treatment period, indicated that a daily dose of 100 μg. of diethylstilbestrol (by injection) could delay tumour growth initiation, although significant increases in the mean latent periods were not observed. The trend was substantiated by following experiments in which the treatment period was extended until after tumour growth was well established in the control groups (Series F9–A, F12, 4 F3). For both males and females, no tumour growth occurred during the treatment period and for some weeks after injections were discontinued. Table III gives the data for a few representative experiments.

TABLE III.—*Effect of High Doses of Diethylstilbestrol Injected from the Time of Tumour Implantation on the Growth of Transplanted Fibroadenomata in Rats.*

| Series. | Treatment. | Sex. | Number of rats. | Number of takes. | Mean latent period (days). | Mean growth period (days). |
|---|---|---|---|---|---|---|
| F9–A | Stilbestrol* | F | 10 | 7 | 123± 4·3‡ | 49± 7·0 |
| | Controls | F | 10 | 7 | 41± 3·6 | 35± 2·0 |
| | Stilbestrol* | M | 10 | 6 | 151±10·6‡ | 53± 6·4 |
| | Controls | M | 10 | 8 | 59± 6·5 | 63±11·5 |
| F12 | Stilbestrol† | F | 7 | 4 | 101± 7·7‡ | 38± 3·2 |
| | Controls | F | 10 | 7 | 42± 6·1 | 39± 3·9 |

* Stilbestrol, 400 μg. every 2nd day for 52 days after tumour implantation.
† Stilbestrol, 200 μg. every 2nd day for 58 days after tumour implantation.
‡ $P < 0 \cdot 01$, treated versus controls.

When administered orally in the drinking water, the daily consumption of diethylstilbestrol was approximately 50 μg. In neither females or males was this amount sufficient to delay the initiation of tumour growth. However, for the females (Series F6) tumour growth was arrested or slowed in 5 of 8 tumourbearing rats. The remaining 3 tumours appeared unaffected. The mean growth-period for this group was significantly longer than the controls.

The growth results for male rats were less easily evaluated. Two groups of 8 rats in the same series (Series F8) received diethylstilbestrol orally. One of these groups received, in addition, injections of a crude beef anterior pituitary preparation (to be described in a later section). Only 2 out of 8 tumours in the group receiving stilbestrol alone showed growth retardation, while the growth rate was markedly reduced in 6 of 8 tumours of the group receiving both estrogen and the anterior pituitary preparations. Since, as it will be shown, the anterior pituitary preparation alone can stimulate the growth of mammary fibro-adenomata in male rats, it was considered unlikely that this product produced an additive inhibiting action with high doses of diethylstilbestrol. Rather, in view of this consideration and the apparent all or none action of diethylstilbestrol when administered at this dose level, the unequal response of these two groups has been attributed to change variation alone. Therefore, the results from these two groups have been pooled and presented as a single group. In this case the mean growth period of the tumours of treated rats was significantly longer than the controls, as may be seen from Table IV.

TABLE IV.—*Effect of High Doses of Diethylstilbestrol administered Orally from the Time of Tumour Implantation on the Growth of Transplanted Fibro-adenomata in Rats.*

| Series. | Treatment.* | Sex. | Number of rats. | Number of takes. | Mean latent period (days). | Mean growth period (days). |
|---|---|---|---|---|---|---|
| F6 | Stilbestrol (continuous) | F | 8 | 8 | 31± 3·4 | 113±25·4‡ |
| | Controls | F | 10 | 9 | 43±15·4 | 35± 3·2 |
| F8 | Stilbestrol† | M | 20 | 16 | 51·5±10·0 | 79·4±13·6‖ |
| | Controls | M | 10 | 10 | 45·0± 3·4 | 32·0± 1·6 |

* Drinking water 0·5 mg. per cent diethylstilbestrol, approximate daily intake = 50 μg.
† Stilbestrol, administered for 168 days after tumour implantation.
‡ $P < 0 \cdot 01$, treated versus controls.
‖ $P < 0 \cdot 05$, treated versus controls.

Finally, experiments were undertaken to determine the effect of high doses of estrogen on already growing tumours. Rats in two series (F5–B and F12) were injected with stilbestrol after the tumours had reached a size of approximately 0·5 square inches. Treatment for 3 weeks (100 μg. stilbestrol daily) caused little change in tumour growth rate (Series F5–B). In Series F12 tumour-bearing rats were injected every second day for 60 days with 200μg. of diethyl-stilbestrol. Tumour growth was arrested in 4 rats, while there was no response in the remaining 3. Since some of these animals died before the tumours reached 3 square inches, it was not possible to calculate a mean growth period for this group. The results are presented, therefore, in Fig. 1, which shows tumour areas plotted against time for each tumour in the treated group. As the retarded tumours did not exhibit a rapid recovery after the cessation of treatment, it cannot be ruled out that the tumour growth trend was independent of the treatment. However, no such slow growing tumours occurred in the controls (mean growth period 39 ± 3·9 days) or any other group in this series, a fact which favours the

FIG. 1.—Effect of high doses of diethylstilbestrol on growing fibroadenomata in rats (Series F12).

interpretation that the estrogen was at least partially responsible for the depressed growth of these tumours.

Estrogens administered at the high dose levels given cause considerable body growth retardation and occasionally incur the death of the animal. It was necessary, therefore, to determine whether tumour growth inhibition was merely concurrent with body growth depression or whether it could be attributed more specifically to the estrogen administered. It has been reported (Millar and Noble, 1952) that dietary restriction sufficient to cause body growth depression equal to that observed in stilbestrol treated rats (100 µg. daily) had little or no effect on the growth initiation of fibroadenoma implants, or on the tumour growth rates. In addition, the initiation of tumour growth after cessation of stilbestrol injections (treatment started at time of tumour implantation) could not be delayed further by dietary restriction, sufficient to prevent the host from recovering from the body weight depressing effects of this hormone.

There did not seem to be any alteration in the distribution of the benign variants of the fibroadenoma in animals treated with high doses of stilbestrol. The incidence of fibrosarcomata was unaltered also in these groups, where tumour growth initiation did not occur till after treatment was discontinued. However, for those animals which received stilbestrol during tumour growth there was an increase in the number of sarcomatous transformations, this event being restricted to tumours whose growth had been arrested or retarded during the treatment. The results for female rats are given in Table V.

TABLE V.—*Effect of High Doses of Diethylstilbestrol Administered During Tumour Growth on the Incidence of Fibrosarcomata in Female Rats.*

| Group. | Benign tumours. | Fibrosarcomata. |
|---|---|---|
| Treated (13 rats) | 5 | 8 |
| Controls (19 rats) | 17 | 2 |

$\chi^2$ test, $P < 0\cdot01$.

According to the $\chi^2$ test there is a significant difference in the incidence of fibrosarcomata in the treated and control groups. This data includes the groups receiving stilbestrol by injection after tumour growth initiation and those receiving diethylstilbestrol orally from the time of tumour implantation. The latter groups are included since the treatment period extended well after tumour growth initiation.

With regard to male rats on this regime (Series F8) the results were less conclusive. Eight of 16 tumours became sarcomatous in the treated group, and again, this change was restricted to those tumours showing retarded or arrested growths. However, 3 of 6 tumours in the controls became sarcomatous. It would appear, therefore, that tumours in the male rats of this series were initially more predisposed to become fibrosarcomata, since in the control group the sarcomatous tumours were fairly fast growing and showed a tendency to fibrous tissue development while in the benign state.

(2) *Effect of progesterone.*

Experiments were conducted with female rats in which progesterone was injected alone (5 mg. daily) or with low doses of estrogen for the " limited " treatment period (Series F4–B and F5–C). No change was observed in the growth characteristics or morphology of the tumours of treated rats.

(3) *Effect of androgen.*

Testosterone proprionate injected into female rats for the " limited " treatment period (1 mg. daily) did not affect the growth or morphology of fibroadenoma implants (Series F4–B and F5–C). However, when the duration of treatment was extended (106 days after tumour implantation) there was an indication of slight tumour growth inhibition (Series F10–A and F11). Due to the death of some animals before the tumours had reached 3·0 square inches, mean growth periods could not be calculated.

Data regarding tumour morphology was scant, but there was a trend towards increased fibrous tissue development in the tumours of testosterone treated rats.

The results in this section are inadequate for any independent conclusions to be drawn. However, when considering the findings of Heiman and Mohs for the action of androgens on the fibroadenoma which were outlined earlier, the trends observed may be significant.

(4) *Effect of cortisone.*

One experiment in Series F5–B was carried out to determine the effect of cortisone on transplanted fibroadenomata in female rats. Cortisone acetate in aqueous suspension was injected for a period of 3 weeks at a dose level of 5 mg.

daily. The treatment was started after the tumours had reached an initial size ranging from 0·6 to 1·4 square inches. All tumours reached a size of 3·0 square inches before the treatment period was completed. There was no difference in the mean growth periods of treated and control groups. Cortisone treatment caused considerable body weight loss, showing again that body growth depression need not slow the growth of transplanted fibroadenomata. Cortisone treated rats were not killed for 4 or more weeks after injections were discontinued. The tumours showed a normal morphology at this time.

(5) *Effect of anterior pituitary preparations.*

Three pituitary preparations were tested for their effect on the growth and morphology of the transplanted fibroadenoma. These were :

(1) a bovine anterior pituitary saline suspension ;
(2) a sheep pituitary extract ;
(3) a biologically pure growth hormone preparation obtained from Armour Laboratories Inc.

(1) The anterior lobes were dissected from frozen whole beef pituitaries. The required amount of anterior pituitary substance was weighed, ground with sand in a mortar and mixed with physiological saline in the proportions of 1 g. of anterior pituitary to 5 ml. of physiological saline. This was mixed well, centrifuged and the supernatant plus the fine sediment at the top of the residue taken off and refrigerated. No more than a week's supply was prepared at one time. This material was injected subcutaneously in doses of 0·5 ml. daily or 1·0 ml. every second day, this being equivalent to 100 and 200 mg. respectively of anterior pituitary substance.

Biological assays have indicated the presence of growth hormone and prolactin but little gonadotrophic activity in this preparation.

(2) A sheep pituitary extract was prepared in this laboratory over 15 years ago with the purpose of obtaining prolactin. No details, however, are available for the method of extraction. The product was a crude one and it was considered that potentially it contained the activity of many of the pituitary hormones. Biological essays indicated that there was prolactin but no growth hormone activity in the extract. Gonadotrophic activity was not determined. The material was injected subcutaneously in a neutralized water suspension at a dose level of 25 mg. daily.

(3) The growth hormone was a water soluble product which, although not chemically pure, was reported to be free of all other biologically active components of the pituitary. Daily doses of 0·5 mg. were administered in water solution by subcutaneous injection.

The beef anterior pituitary preparation was administered to female, male, and ovariectomized female rats (Series F5–C, F6, F8, F11 and 4F–3). In addition, female castrates and males were treated with both the anterior pituitary and diethylstilbestrol (5 $\mu$g. daily or 10 $\mu$g. every second day), (Series F6 and F9–B). The sheep pituitary extract and the growth hormone were tested in single experiments with female rats (Series F9–A and F–12). Representative results are given in Table VI

Both beef anterior pituitary and sheep pituitary preparations stimulated the growth of the fibroadenomata. The former was active in males and castrated

TABLE VI.—*Effect of Various Pituitary Preparations on the Growth of Transplanted Fibroadenomata in Rats.*

| Series. | Treatment.* | Sex. | Number of rats. | Number of takes. | Mean latent period (days). | Mean growth period (days). |
|---|---|---|---|---|---|---|
| F6 | A.P.S.,† limited | F | 8 | 8 | 22± 1·1 | 21± 1·0‡ |
| | Controls, intact | F | 10 | 9 | 43±15·4 | 35± 3·2 |
| | Ovariectomy + A.P.S., limited | F | 8 | 8 | 24± 1·0 | 23± 1·9‡ |
| | Ovariectomy + (A.P.S. and Stilbestrol), limited | F | 8 | 8 | 21± 1·2‖ | 27± 1·4‡ |
| | Ovariectomized controls | F | 8 | 7 | 29± 2·9 | 37± 1·6 |
| F11 | A.P.S., continuous | F | 10 | 5 | 46± 2·2 | 43± 7·1 |
| | Controls, intact | F | 10 | 7 | 61± 6·2 | 56±21·9 |
| | Ovariectomy + A.P.S., continuous | F | 10 | 5 | 56± 5·7 | 59±15·1 |
| | Ovariectomized controls | F | 10 | 0 | — | — |
| F8 | A.P.S., continuous | M | 10 | 9 | 38± 2·2 | 26± 2·0‖ |
| | Controls | M | 10 | 10 | 45± 3·4 | 32± 1·6 |
| F9–B | A.P.S., continuous | M | 10 | 4 | 80±14·6 | 32± 3·2 |
| | A.P.S. + stilbestrol, continuous | M | 10 | 8 | 69± 4·6 | 78±13·6 |
| | Controls | M | 10 | 0 | — | — |
| F9–A | Sheep pituitary extract, continuous | F | 10 | 8 | 34± 1·7 | 29± 0·8‖ |
| | Controls | F | 10 | 7 | 41± 3·6 | 35± 2·0 |
| F12 | Growth Hormone, continuous | F | 10 | 8 | 48± 5·1 | 43± 9·7 |
| | Controls | F | 10 | 7 | 42± 6·1 | 39± 3·9 |

* Treatment, for dose levels see description in text.
† A.P.S., beef anterior pituitary suspension.
‡ $P < 0{\cdot}01$, treated versus controls.
‖ $P < 0{\cdot}05$, treated versus controls.

females as well as in intact females. During the course of some of the experiments using the beef anterior pituitary mixture, penicillin was administered for a short period to allay infection induced by the injected material. This treatment proved to be satisfactory and did not appear to influence the tumour growth. The action of beef anterior pituitary was not evident in all experiments with intact females (Series F9–B and F11). In these cases, however, the individual variation was extreme in both control and treated animals, a condition which may have masked any existing response. Stilbestrol did not appear to augment the anterior pituitary stimulation of fibroadenomata in castrated females or in males.

In the single experiment conducted, there was no evidence of a response to pure growth hormone.

There was no change in the morphology of the benign tumours or the incidence of fibrosarcomata with any of the treatment regimes given.

### DISCUSSION.

Although the fibroadenoma is more successfully propagated in the female host, estrogen administered in small doses to intact rats caused no change in tumour growth or morphology, except in one early experiment (F3). This one exception may be attributed to chance variation or can be interpreted as evidence of a greater responsiveness in early tumour generations. This possibility is supported by the findings of Heiman and Mohs, which were reviewed earlier, but is not upheld by the later work using third generation tumours of Lines 3F and 4F.

In male rats, however, small amounts of exogenous estrogen produced tumour growth and morphology comparable to that of female controls. This response supplies further proof for the need of minimal estrogen levels for maximum growth of the mammary fibroadenoma in rats. Depression of the male sex hormone may also be a factor involved, particularly with regard to the increased epithelial development of tumours in estrogen treated males. The inhibition of tumour epithelium by testosterone was postulated in the preceding paper (Millar and Noble, 1954), when it was observed that tumours were more fibrous in male rats than in female controls, but were unchanged morphologically in castrated females. Experiments with administered androgens tend to support this contention. Mohs (1941) reported that prolonged injections of small doses of testosterone brought about a progressive loss of tumour epithelium but no change in growth rate. Using a higher dose level and shorter treatment period, in this laboratory the tumours tended to be more fibrous and showed only slight growth inhibition. Heiman (1940*a* and 1943) reported a similar but more dramatic response to the action of testosterone on both growth rate and epithelial development. Dose levels, not clearly defined, were probably intermediate to those used by Mohs (1941) and those used in this laboratory. High susceptibility to change seems typical of the tumours studied by this worker. This may be a characteristic of the strain of rat used, for the Sprague-Dawley strain employed by us was also used by Mohs.

The negative response of fibroadenomata to administered progesterone reported herein, as opposed to the marked inhibition observed by Heiman (1940*a* and 1943) offers another example of varied susceptibility. Although in our experiments, progesterone treatment was for the 4-week period only, the relatively undramatic effect of large doses of testosterone in intact females makes a marked response to progesterone rather difficult to envision even if the treatment period had been prolonged.

Inhibition of the growth of transplanted mammary fibroadenomata produced by high doses of estrogen has not been reported by other workers. To the authors' knowledge Emge (1939) is the only worker to use similar high levels of estrogens in studies on mammary fibroadenomata. His experiments were designed to study the effect of such treatment on the incidence of carcinoma in these benign tumours. Marked epithelial hyperplasia, but no evidence of carcinomatous transformation was observed. It is assumed that the treatment occurred after tumour growth-initiation. No reference is made to alterations in tumour growth rate. A systematic study of changes in tumour morphology during treatment has not been carried out in this laboratory, but examination of tumours from a few rats which died during treatment periods, gave no indication of the pronounced epithelial response observed by Emge (1939). This again demonstrates the limited capacity of the epithelium of the tumours observed by us to respond to environmental changes without the physiological range.

Considerable speculation could be devoted to the mode of action of stilbestrol on the growth of fibroadenomata. The most obvious possibility, i.e., that of a nonspecific reflection of body growth depression, has been ruled out by paired feeding experiments. The fibroadenoma may be responding like normal immature mammary tissue, for it has been shown that mammary gland development in young rats is depressed by large doses of estrogen (Astwood, Geschickter and Rausch, 1937). It is well known that excessive estrogen will inhibit the growth of some malignant neoplasms in rats and mice (Eisen, 1941 ; Nathanson and Salter, 1939) and in humans such action is well exemplified by the use of estrogen therapy for breast cancer. The variability of response in humans (Nathanson and Kelley, 1952) brings to mind the all or none response obtained with stilbestrol administration to rats bearing growing fibroadenomata. It is difficult to assess to what extent the benign fibroadenoma can be compared with malignant growths or to normal tissue with regard to the mechanism of response to estrogen excesses. Nevertheless, the hormonal imbalance created, with the resulting depression of anterior pituitary function, very likely plays an important part in the inhibition of the benign fibroadenoma in other types of breast tumours and as well in normal breast tissue.

From a therapeutic point of view the desirability of such a response in the fibroadenoma is questionable, since the incidence of malignant transformations was increased in the groups treated during tumour growth, this event being coincident with tumour growth inhibition. In the preceding paper (Millar and Noble, 1954) it was shown that the change to fibrosarcoma was restricted to slow growing fibroadenomata and it was proposed that the fibrosarcomatous transformation was instigated in an effort to overcome the action of some intrinsic inhibitory agent. Apparently, contradictory to this theory, was the lack of an increased incidence of fibrosarcomata in the tumours of rats injected with high doses of stilbestrol from the time of tumour implantation, where artificially prolonged latent periods of over 200 days were observed. In view of this more dramatic growth response it is difficult to understand why stilbestrol administration during tumours growth should increase the incidence of fibrosarcomata. Possibly the treatment or the resulting growth inhibition is more effective in bringing about a fibrosarcomatous transformation in established tumours which have been actively growing than in the temporarily dormant implant.

The findings reported for the action of anterior pituitary preparations on the mammary fibroadenoma are of particular interest in view of the currently increasing evidence of the part played by pituitary hormones in carcinogenesis (Moon, Simpson, Li and Evans, 1950 ; Moon, Simpson and Evans, 1952 ; Noble and Walters, 1954).

The identity of the pituitary component or components which stimulate the growth of the fibroadenoma is not known. Since both the beef and sheep pituitary preparations used were crude products, the response was probably a result of the integrated action of several pituitary hormones. However, if a single hormone was responsible for the increased tumour growth rates observed, prolactin would seem to be the most likely possibility. Gonadotrophic activity was low in the beef pituitary preparation, as was growth hormone activity in the sheep pituitary extract. Negative results with purified growth hormone further justifies its elimination. Prolactin activity was high in both crude preparations. Adrenocorticotrophin and thyrotrophin were probably present and cannot be eliminated as possible active factors, but one is inclined to favour prolactin in view of its important role in mammary tissue development and lactation (Folley, 1952).

The lack of response to purified growth hormone confirms the findings of Heiman and Krehbiel (1936). However, no synergism was noted between estrogen and the beef anterior pituitary preparation, while these workers reported that estrogen plus gonadotrophin or growth hormone stimulated tumour growth although none of the individual products were active in intact rats.

In conclusion, a review of the findings reported herein and elsewhere leads to the realization that in the mammary fibroadenoma one is dealing with a tissue possessing both normal and neoplastic properties. Being of mammary origin these tumours frequently respond to hormones involved in mammary gland development, but the type and degree of response often differs widely from that expected of normal tissue. Thus, testosterone which stimulates alveolar development in mammary tissue, depresses the epithelium of transplanted fibroadenomata. Alternately, estrogen at physiological levels which normally stimulate epithelial development alone promotes the growth of these predominantly connective tissue tumours. Estrogen excesses may or may not increase glandular tissue in these tumours, the variation between tumour lines and strains of rats being marked in this respect. Finally, cortisone which causes the breakdown of some collagen connective tissues, is ineffective in altering the growth characteristics of the mammary fibroadenoma.

SUMMARY.

1. The administration of small doses of diethylstilbestrol to female rats did not alter the growth characteristics or morphology of transplanted mammary fibroadenomata. However, tumour growth rate and epithelial development in similarly treated male rats was increased to equal that of female controls.

2. High levels of diethylstilbestrol (100–200 $\mu$g. daily) prevented tumour growth initiation when administered from the time of tumour implantation. If treatment was commenced after tumour growth initiation, the response was less uniform, but if any, was one of growth retardation or arrest. The incidence of fibrosarcomata was increased in the latter group and this event was restricted to the fibroadenomata showing slowed or arrested growth.

3. Testosterone proprionate produced slight tumour growth inhibition and an indication of depressed tumour epithelial development. Experimental findings were inconclusive but the results of others support the trends observed.

4. Progesterone and cortisone in the amounts and for the duration administered did not alter the growth rate or morphology of transplanted fibroadenomata in female rats.

5. Crude beef and sheep pituitary preparations stimulated tumour growth in intact female rats. The former was also active in males and castrated females. The simultaneous administration of small doses of estrogen did not augment the action of the beef pituitary preparation. Biologically pure growth hormone did not alter the growth or morphology of the transplanted fibroadenomata in female rats.

This work has received continuous financial support from the National Cancer Institute of Canada.

Miss Bette Byrns rendered valuable technical assistance in these experiments.

REFERENCES.

Astwood, E. B., Geschickter, C. F., and Rausch, E. O.—(1937) *Amer. J. Anat.*, **61**, 373.

Eisen, M. J.—(1941) *Cancer Res.*, **1**, 457.

EMGE, L. A.—(1939) *Surg., Gynec. and Obstet.*, **68**, 472.
*Idem* AND MURPHY, K. M.—(1938) *Proc. Soc. exp. Biol., N.Y.*, **37**, 620.
*Iidem* AND SCHILLING, W.—(1938) *Ibid.*, **38**, 21.
FOLLEY, S. J.—(1952) *Recent Progr. Hormone Res.*, **7**, 107.
HEIMAN, J.—(1940*a*) *Amer. J. Cancer*, **39**, 178.—(1940*b*) *Ibid.*, **40**, 343.—(1943) *Cancer Res.*, **3**, 65.
*Idem* AND KREHBIEL, F.—(1936) *Amer. J. Cancer*, **27**, 450.
MILLAR, M. J., AND NOBLE, R. L.—(1952) *Cancer Res.*, **12**, 282.—(1954) *Brit. J. Cancer*, **8**, 485.
MOHS, F. E.—(1940) *Amer. J. Cancer*, **38**, 212.—(1941) *Cancer Res.*, **1**, 151.
MOON, H. D., SIMPSON, M. E., AND EVANS, H. M.—(1952) *Science*, **116**, 331.
*Idem*, SIMPSON, M. E., LI, C. H., AND EVANS, H. M.—(1950) *Cancer Res.*, **10**, 297.
MURPHY, K. M., SCHILLING, W., AND EMGE, L. A.—(1938) *Proc. Soc. exp. Biol., N.Y.*, **39**, 298.
NATHANSON, I. T., AND KELLEY, R. M.—(1952) *New Engl. J. Med.*, **246**, 135.
*Idem* AND SALTER, W. T.—(1939) *Arch. Path.*, **27**, 828.
NOBLE, R. L., AND WALTERS, J. H.—(1954) *Proc. Amer. Ass. Cancer Res.*, **1**, 35.